# Treatment strategy for acute myocarditis in pediatric patients requiring emergency intervention

**DOI:** 10.1186/s12887-023-04200-0

**Published:** 2023-08-05

**Authors:** Yuka Murakoshi, Kenji Hoshino

**Affiliations:** Division of Pediatric Cardiology, Saitama Children’s Medical Center, 1-2 Shintoshin, Chuo-Ku, Saitama City, Saitama, 330-8777 Japan

**Keywords:** Acute myocarditis, Emergency intervention, Extracorporeal membrane oxygenation, Temporary pacing, Wide QRS complexes

## Abstract

**Background:**

Patients with acute myocarditis present with a wide range of symptoms. Treatment strategies for pediatric patients with circulatory failure comprise extracorporeal membrane oxygenation (ECMO), emergency temporary pacing, and pharmacotherapy. However, they remain controversial. ECMO is an effective treatment but gives rise to several complications; the goal is therefore to avoid excessive treatment as much as possible. We aimed to evaluate the importance of electrocardiogram findings in differentiating severity and establish an appropriate treatment strategy in pediatric patients with acute myocarditis who required emergency interventions.

**Methods:**

This retrospective study enrolled pediatric patients admitted to and treated in our hospital for acute myocarditis between April 1983 and December 2021. Patients were retrospectively divided into whether circulatory failure occurred (ECMO or temporary pacing was needed; emergency intervention group) or not (pharmacotherapy alone).

**Results:**

Of the 26 pediatric patients, 11 experienced circulatory failure while 15 did not. In the circulatory failure group, six patients were treated with ECMO (ECMO group) and five patients with temporary pacing (pacing group). In the pacing group, all patients were diagnosed with complete and/or advanced atrioventricular block (CAVB and/or advanced AVB) and narrow QRS. Furthermore, these patients improved only with temporary pacing and pharmacotherapy, without requiring ECMO. Wide QRS complexes (QRS ≥ 0.12 ms) with ST-segment changes were detected on admission in five of six patients in the ECMO group and none in the pacing group (P = 0.015). Although all patients in the ECMO group experienced complications, none did in the pacing group (*P* < 0.008).

**Conclusions:**

Regarding emergency intervention for acute myocarditis, ECMO or temporary pacing could be determined based on electrocardiogram findings, particularly wide QRS complexes with ST-segment changes on admission. It is important to promptly introduce ECMO in patients with wide QRS complexes with ST-segment changes, however, patients with CAVB and/or advanced AVB and narrow QRS could improve without undergoing ECMO. Therefore, excessive treatment should be avoided by separating ECMO from temporary pacing based on electrocardiogram findings on admission.

## Introduction

Acute myocarditis refers to inflammation of the myocardium, which is characterized by leukocyte infiltration, followed by fibrosis and necrosis. It is rare, accounting for 0.05% of hospitalized pediatric patients [1]. It also has a broad range of clinical manifestations ranging from mild symptoms, such as gastroenteritis or upper respiratory tract infection and chest pain, to life-threatening arrhythmias or severe cardiogenic shock [2]. Acute myocarditis can generally be divided into fulminant and non-fulminant forms based on the severity of clinical presentation. Patients with circulatory failure may require mechanical circulatory support such as extracorporeal membrane oxygenation (ECMO) and temporary pacing. However, some cases could improve with pharmacotherapy only, including intravenous immunoglobulin, inotropes, and antiarrhythmic medication for vasoactive support, without clinically relevant residual damage [3–5]. Although ECMO is an effective treatment, it is associated with several complications, such as hemorrhage, neurological sequelae, and intracardiac thrombosis, especially in pediatric patients. Therefore, it is crucial to determine an accurate indication for ECMO in cases where the benefits outweigh the potential harm. However, owing to the wide variety of clinical presentations of acute myocarditis, clinical practice guidelines regarding its evaluation and treatment are still lacking [6]. Wide QRS complexes (QRS ≥ 0.12 ms) with ST-segment changes on electrocardiogram (ECG) indicate severe damage to the cardiomyocytes, secondary to intraventricular conduction delay and the cardiac conduction system block. Some studies have been conducted to assess the severity of acute myocarditis based on wide QRS complexes, which is controversial. Details of the clinical usefulness of ECG data to determine the severity of the disease are limited [7–9]. Although making accurate diagnoses and treatment decisions based on assessing the severity of acute myocarditis is essential, there is a dearth of clear guidelines [10].

This study aimed to evaluate the importance of ECG data on admission in differentiating the severity of acute myocarditis and establish an appropriate treatment strategy in pediatric patients with acute myocarditis requiring emergency intervention.

## Methods

### Patients and methods

We retrospectively reviewed the medical records of pediatric patients with acute myocarditis treated at Saitama Children’s Medical Center between April 1983 and December 2021. Acute myocarditis was diagnosed based on clinical symptoms and laboratory findings, including blood tests, chest radiography, and echocardiography, per the guidelines for diagnosing and treating acute myocarditis in Japan [6].

In general, fulminant myocarditis is defined as cases treated with ECMO or that cannot improve without ECMO treatment [11, 12], but some cases in fulminant myocarditis group do not necessarily require ECMO. Therefore, in this study, we did not use the fulminant/non-fulminant classification but the classification described below.

First, the patients were divided based on whether circulatory failure occurred (ECMO or temporary pacing was needed for recovery from circulatory failure; emergency intervention group) or not (pharmacotherapy alone). Circulatory failure in our study was defined as an inability to maintain circulation due to circulatory collapse and Stokes–Adamas seizures by atrioventricular block (AV block) according to the diagnostic criteria of the Myocardial Infarction Research Units of the National Heart and Lung Institute [13].

Second, in the emergency intervention group, patients were divided into the ECMO and temporary pacing groups. The two groups were compared by reviewing the medical records of patients. These data included clinical and anthropometric parameters such as age, sex, time from the first symptom to visiting a referring physician or our center, vital signs, ECG on admission, treatment, outcome, complications, and number of days in the hospital. The ECG on admission focused on wide QRS, defined as QRS ≧0.11 ms for up to elementary school students and QRS ≧ 0.12 ms for junior high school students, based on the Guideline for Selecting Candidates for Secondary Screening of Heart Disease in Schools (JSPCCS2019) [14]. Hypotension on admission was defined according to the 2020 Japanese Guidelines for Management of Sepsis and Septic Shock practice 2020 [15].

We obtained informed consent, and this study was approved by the Ethics Committee of Saitama Children’s Medical Center (approval number: 2021–06-019).

### Statistical analysis

All statistical analyses were performed using EZR [16]. Fisher’s exact test or the Mann–Whitney U test was used for intergroup comparisons. Statistical significance was set at *P* < 0.050.

## Results

**Fig. 1:**
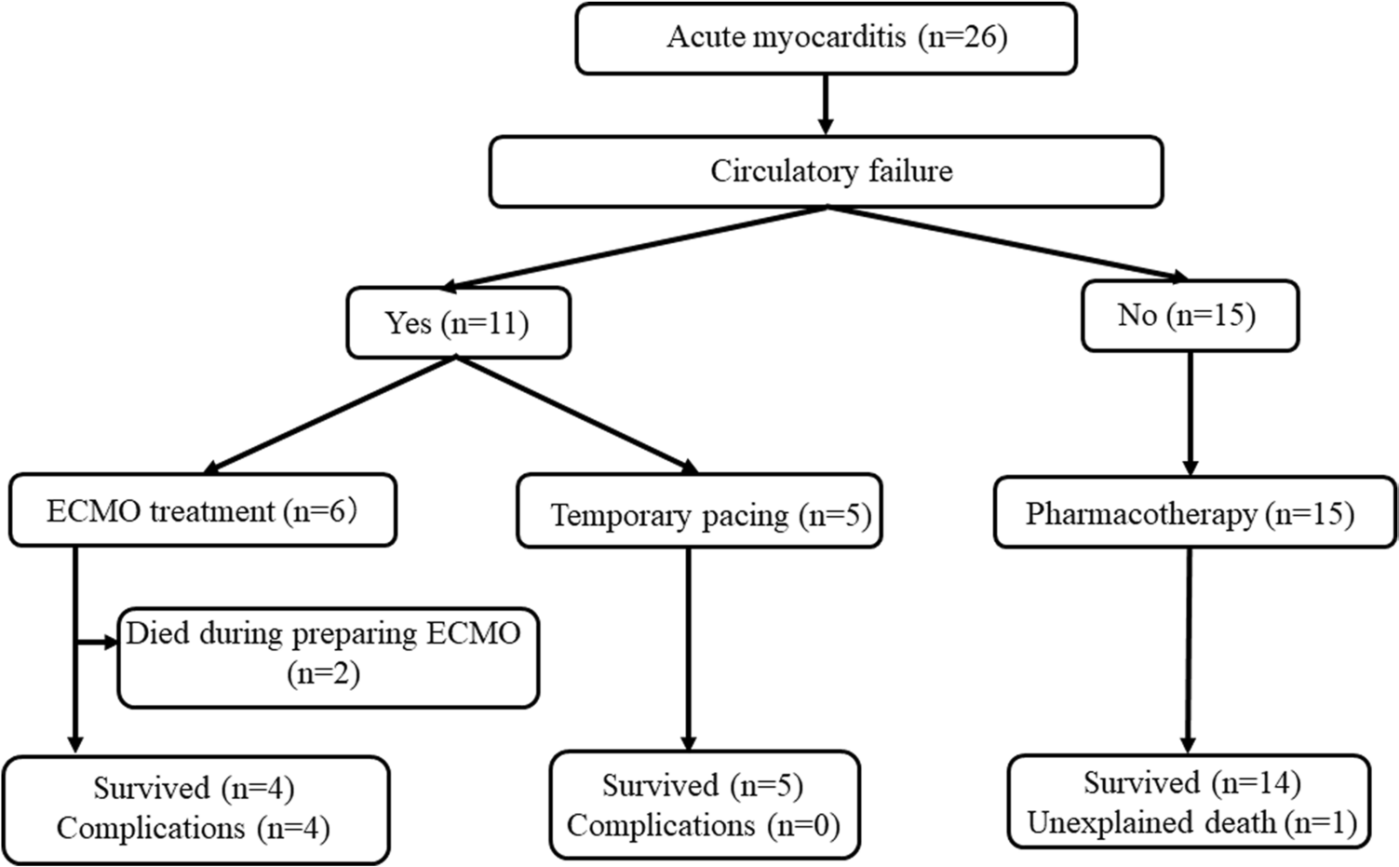
Flowchart and treatment outcomes of pediatric patients with acute myocarditis. Abbreviations: AVB, atrioventricular block; CAVB, complete atrioventricular block; ECG, electrocardiogram; ECMO, extracorporeal membrane oxygenation

Figure 1 describes the workflow of this study and treatment outcomes of pediatric patients with acute myocarditis. A total of 26 pediatric patients (mean age: 5.5 years, range: 0–15 years, 10 boys) with acute myocarditis were included in this study. The demographic data of all 26 pediatric patients is as follows; LV EF; median 58% (range 10–75%), duration of hospitalization; median 20 days (ranges 2–102 days), and laboratory findings such as lactic acid 1.9–12.5 mmol/L, OT/PT 24–41/13–27 s, BUN/Cr 41–70, cardiac enzyme (CK 33–17,378 U/L,CKMB 2–9%, TroI 599–6460 pg/mL), and BNP 80–3141 pg/mL. An etiology of myocarditis was determined in only one 1 of 26 cases, with parvovirus B19.

First, the patients were divided based on whether circulatory failure occurred—11 patients needed emergency intervention due to circulatory failure. However, 15 patients were treated with only pharmacotherapy. Second, the patients in the emergency intervention group were divided into two groups according to the ECG findings on admission, wide QRS complexes with ST-segment changes (rise or fall of 0.1 mV or more from J point, at least one location)—five patients had wide QRS complexes with ST-segment changes on admission. Of 11 patients required emergency intervention, six patients were treated with ECMO (the ECMO group), and five were treated with temporary pacing (the temporary pacing group). Table 1 compares the clinical characteristics and treatment outcomes between the ECMO and temporary pacing groups. There were no significant differences between the patients in the ECMO and temporary pacing group in age, sex, time from the first symptom to visiting the referring physician or our center, vital signs on admission, outcome, or duration of hospitalization. Wide QRS complexes with ST-segment changes on ECG were significantly more prominent in the ECMO group than in the temporary pacing group (*P* = 0.015). Concomitant medications were as follows in the ECMO group: one case under Ancalon continuous infusion; in the temporary pacing group: one case under disopyramide bolus infusion and one case during continued adrenaline support. All patients treated with ECMO had complications such as peroneal nerve palsy and intracranial disease (*P* = 0.008).

**Table 1:** A comparison of the clinical characteristics and treatment outcomes between ECMO and temporary pacing groups

	ECMO group (*n* = 6)	Temporary pacing group (*n* = 5)	*P* value
Age in years (median, ranges)	6.7 ± 5.0 (5, 2–15)	9.2 ± 2.3 (10, 6–12)	0.268
Sex (male/female)	2 / 4	0 / 5	0.456
Time from the first symptom to the referring doctor's visit (days): median (ranges)	2.5 (2–6)	3.0 (2–4)	0.922
Time from first symptoms to our hospital visit (days): median (ranges)	3.0 (2–7)	4.0 (3–5)	0.508
Decreased blood pressure on admission (case)	3 (50%)	3 (60%)	1.000
Wide QRS complexes and ST-segment changes on admission (case)	5 (83%)	0 (0%)	0.015
Outcome Survived / Died (case)	4 / 2	5 / 0	0.455
Complication caused by treatment (case)	4 (100%)	0 (0%)	0.008
Hospitalization duration (days) [mean]	29.5	20	1.000

Abbreviation: *ECMO* Extracorporeal membrane oxygenation

Myocardial biopsies and other imaging studies, such as cardiac magnetic resonance imaging, were not performed to make a definitive diagnosis.

### Treatment outcomes after emergency intervention

#### Patients treated with ECMO

Five patients already had circulatory collapse at arrival in the ECMO group and underwent immediate ECMO. Two patients presented with sustained ventricular tachycardia and died of cardiogenic shock while preparing for ECMO.

**Fig. 2:**
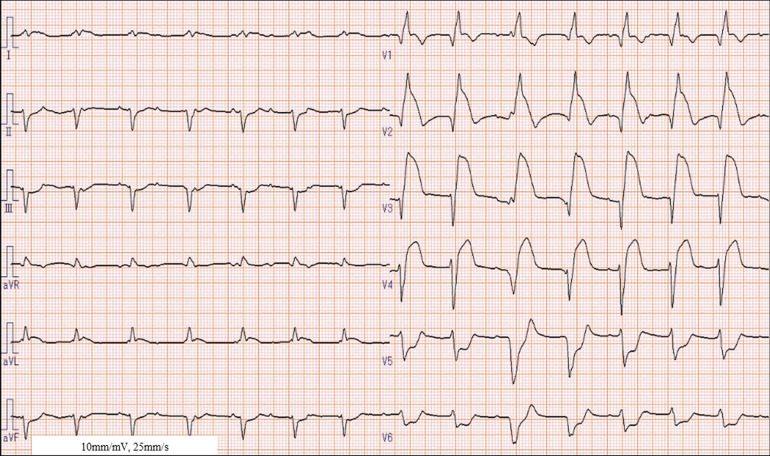
Electrocardiogram on admission in a 6-year-old patient treated with ECMO. Atrial rate: 120–130 bpm, Ventricular rate: 80–90 bpm, complete atrioventricular block, QRS ≥ 0.11 ms, extensive ST-segment changes

**Fig. 3:**
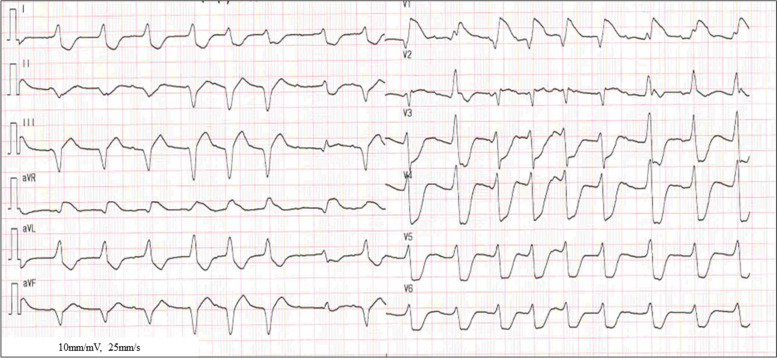
Electrocardiogram on admission in a 2-year-old patient treated with ECMO. Atrial rate: 150 bpm, Ventricular rate: 80–100 bpm, complete atrioventricular block, QRS ≥ 0.11 ms, extensive ST-segment changes

Wide QRS complexes with ST-segment changes on ECG were observed in five of six patients in the ECMO group. The ECG on admission in the ECMO group is shown in Figs. 2 and 3. A 6-year-old boy presented with a complete atrioventricular block (CAVB) and wide QRS complexes with ST-segment changes (Fig. 2). The patient’s condition improved with ECMO support for 4 days. A 2-year-old girl presented with CAVB and wide QRS complexes with ST-segment changes and received ECMO support for 9 days (Fig. 3). Only one patient in the ECMO group had a narrow QRS score. The patient had CAVB and mildly decreased left ventricular ejection fraction (LVEF) and was transferred to our hospital for ECMO treatment. The patient’s condition improved with ECMO support for 1 day. However, ECMO may have been unnecessary, and the patient’s condition may have improved with only pacing and pharmacotherapy.

Among the four patients treated with ECMO for 1 to 9 days, all experienced ECMO-induced complications (*P* < 0.05), such as peroneal nerve palsy (three cases) and intracranial disease (one case). However, all patients improved with rehabilitation.

### Patients treated with temporary pacing

**Fig. 4:**
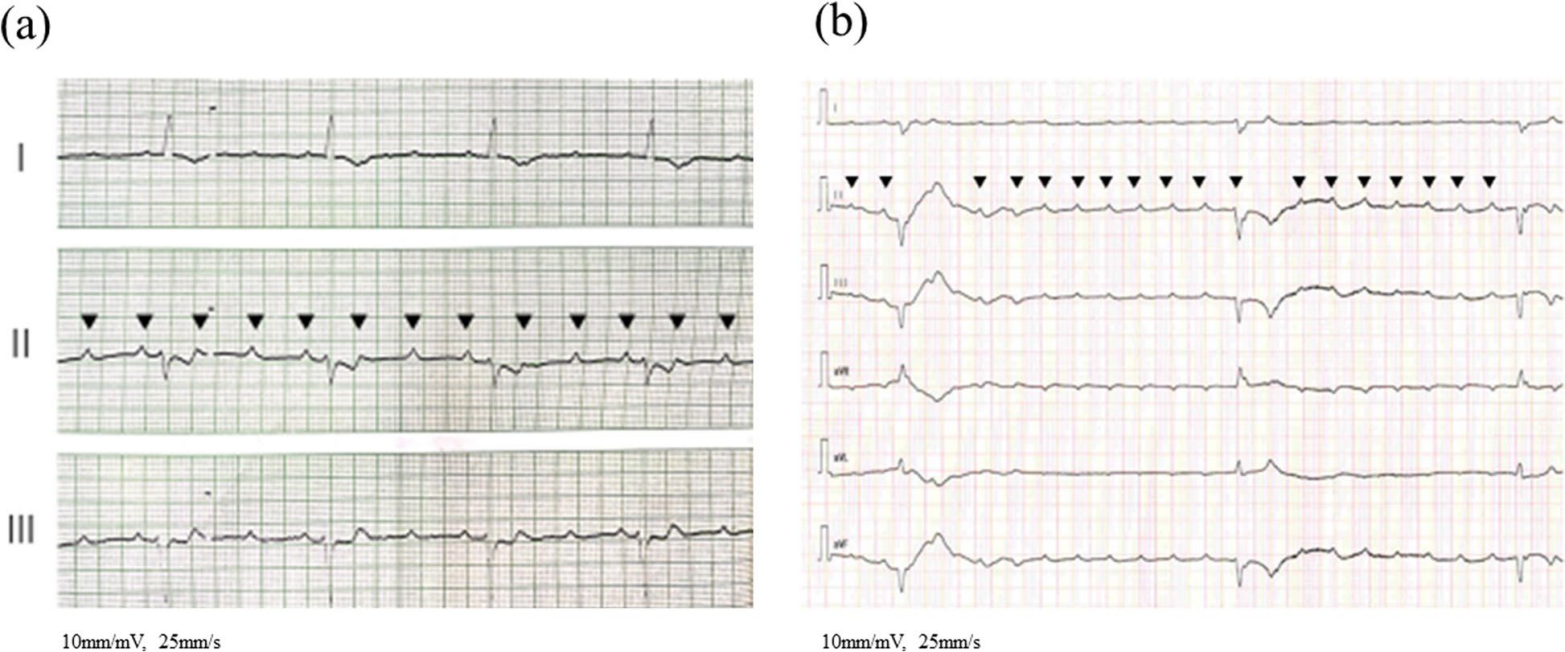
Electrocardiogram on admission in patients treated with temporary pacing. **a** A 10-year-old patient. Atrial rate: 120–130 bpm, Ventricular rate: 40 bpm, advanced atrioventricular block, QRS < 0.11 ms, no ST-segment changes. **b** An 8-year-old patient. Atrial rate: 140–150 bpm, Ventricular rate: 10–20 bpm, advanced atrioventricular block, QRS < 0.11 ms, no ST-segment changes. < Abbreviations > bpm: beats per minuets

Table 2 shows the clinical characteristics of the pediatric patients in the temporary pacing group. All five patients experienced convulsions and syncope (Stokes–Adams seizures) within a few days after presenting with initial symptoms. On admission, none of the five patients had circulatory collapse, and all had CAVB and/or advanced AVB and narrow QRS (Fig. 4). They were treated with pacing support for 1 to 6 days and improved without complications. Pacing was effective in all patients; no patients needed a permanent pacemaker.

**Table 2:** Pediatric patients’ clinical characteristics in the temporary pacing group

Case No	Age (years)	LVEF (%)	VT	Atrial rate (bpm)	Recovery time (day)	Follow-up ECG
1^a^	12	10^b^	-	140	4	CRBBBs
2	10	61	-	130	3	NSR
3	10	36	-	120	6	NSR
4	8	64	-	140	1.5	NSR
5^c^	6	36	+	100	1	NSR

*Abbreviations*: *CRBBBs* complete right bundle branch block, *ECG* electrocardiogram, *LVEF* left ventricular ejection fraction (measured by *M*-mode echocardiography), *NSR* normal sinus rhythm, *VT* ventricular tachycardia

^a^Under Disopyramide bolus infusion

^b^LVEF improved after pacing

^c^During continuous Adrenaline support

### Patients treated with pharmacotherapy

In the non-emergency intervention group, 14 of 15 patients improved, and one patient died due to unexplained tamponade 2 months after disease onset.

## Discussion

The optimal treatment strategy for acute myocarditis in pediatric patients who require emergency intervention, such as ECMO or temporary pacing, remains controversial. Based on this study of pediatric patients with acute myocarditis who underwent emergency intervention, ECMO or temporary pacing could be classified according to ECG findings on admission, particularly wide QRS complexes with ST-segment changes.

The relationship between wide QRS complexes and the severity of acute myocarditis has been reported [17, 18]. Acute myocarditis causes inflammation that affects the ventricular myocardium and cardiac conduction system, inducing systolic cardiac dysfunction and various arrhythmias. Wide QRS complexes are caused by intraventricular conduction delay or bundle branch block. Morgera et al. reported that abnormal QRS complexes were associated with more severe decreases in LVEF and a higher frequency of hypertrophy and fibrosis based on histological examination [17]. We consider that wide QRS complexes are not sinus rhythm but accelerated ventricular rhythm. The myocardial damage is wide and severe, resulting in wider QRS complexes with ST-segment changes, and the narrow QRS complexes are junctional escaped rhythms. The myocardial damage is not severe, resulting in no wide QRS. Although its molecular mechanism is unknown, a recent study showed Cx43, a principal conductor of intercellular current in the ventricle, is one of the molecular determinants for the prolongation of QRS duration [19].

In our study, all five patients treated with ECMO (except one who did not need ECMO) and no patients in the pacing group showed wide QRS complexes with ST-segment changes on admission. Therefore, ECMO or temporary pacing, as emergency interventions in pediatric patients with acute myocarditis, could be classified based on wide QRS complexes with ST-segment changes on admission. Therefore, this strategy is superior to introduce actively ECMO therapy, based on previous case reports in which patients were saved only by temporary pacing and this study. In addition, I consider this strategy more appropriate than introducing more active ECMO, because narrow QRS complexes suggest no extensive and intense myocardial damage.

In our study, we did not perform myocardial biopsy because of an environment in which it can not be performed at our facility. Therefore, we made a comprehensive diagnosis based on symptoms and various laboratory findings.

### Indication for ECMO

ECMO is generally effective in patients with acute myocarditis with circulatory failure. However, to date, no criteria for ECMO intervention are available. The relationship between the severity of acute myocarditis and ECG findings, including adult cases, has been reported [10, 20–22].

Wide QRS complexes in patients with acute myocarditis were among the earliest identifiable clinical signs and indicators of poor prognosis. Additionally, they represented the only independent factor significantly associated with a fulminant course of acute myocarditis [20–22]. Zhang et al. reported that in adult patients (39 ± 14 years) with acute myocarditis, wide QRS complexes on admission in the severe group were predominantly higher than those in the non-severe group. In addition, in the severe group, the non-surviving group had wide QRS complexes significantly more predominant than those in the surviving group [23].

In our study, circulatory failure and wide QRS complexes with ST-segment changes on admission were predominantly observed in the ECMO group. However, the patients in this group improved rapidly with ECMO. We suggest that wide QRS complexes with ST-segment changes in patients with acute myocarditis might be associated with a high risk of circulatory failure and require ECMO support. Therefore, we recommend the prompt use of ECMO in pediatric patients with wide QRS complexes and ST-segment changes on admission.

### Indication for temporary pacing

Acute myocarditis complicated by CAVB and/or advanced AVB is rare in children. Although there are several pediatric studies on acute myocarditis, patients with CAVB were mainly described in case reports. Moreover, the outcomes of CAVB complicated by myocarditis are variable.

Some studies reported that temporary pacing for 1 to 10 days was effective, although permanent pacemakers were sometimes needed [24–30]. Wang et al. reported that temporary pacing and pharmacotherapy were effective in nine pediatric patients (age range: 10 months to 13 years) with acute myocarditis accompanied with CAVB [24], three of whom had ventricular tachycardia in addition to CAVB, and five had Stokes–Adams seizures. In eight patients, cardiac rhythm returned to a normal sinus rhythm within 3.5 ± 3.3 days (range: 0.5–10 days) with temporary pacing, except for one case with an implanted permanent pacemaker. Batra et al. reviewed 40 pediatric patients (mean age: 10.1 ± 6.1 years) with acute myocarditis complicated by advanced AVB [25]. Of the 38 patients treated with temporary pacing in this study, 27 recovered from AV conduction delay. The average time for recovery from AV conduction delay was 3.3 ± 2.8 days. One study reported that persistent CAVB was 22% in children (mean age: 9.5 ± 4.2 years), in which permanent pacemaker implantation could be considered [31].

In our study, patients with CAVB and/or advanced AVB showed narrow QRS. Therefore, we considered the myocardial inflammation not severe and decided on temporary pacing without ECMO. All patients with CAVB and/or advanced AVB and narrow QRS improved with temporary pacing and pharmacotherapy. In addition, there were no cases in which the QRS width increased over time in patients with narrow QRS complexes on admission. None of the patients required ECMO later owing to ineffective temporary pacing.

We suggest that temporary pacing could be effective, and ECMO might not be required in pediatric patients with CAVB and/or advanced AVB and narrow QRS. Regarding emergency interventions for acute myocarditis, conduction system failure improves during temporary pacing, and hence, ECMO can be avoided in these cases. Therefore, excessive treatment should be avoided by separating ECMO from temporary pacing based on ECG findings on admission.

However, there are cases in which the general condition and laboratory findings change rapidly. In our study, there were not any cases where the electrocardiogram or circulatory status changes after hospitalizationafter, but it is quite possible and we need to consider how to respond to such cases. If such status changes after admission, we follow the same approach, stratify the patient again by ECG findings, and consider therapeutic intervention based on these findings.

### Clinical complications of ECMO

Although ECMO is effective, complications suffer from ECMO such as hemorrhage, thrombus formation, internal cranial hemorrhage, cerebral infarction, and neurological sequelae are common [32]. Neurological sequelae includes subarachnoid hemorrhage, ischemic watershed infarctions, hypoxic-ischemic encephalopathy, unexplained coma, and brain death, which has potentially fatal neurological diagnoses and it is associated with significant increase in mortality [33, 34]. The children have more neurologic complications than adults [35]. Neurological complications are highly variable ranging between 4–37%, and the incidence of major neurologic morbidity in cardiac patients is highest in neonates, specifically, with 7% suffering seizures, 3.5% infarction, and 11% intracranial hemorrhage. Children have slightly lower incidence of seizures and hemorrhage and a slightly higher incidence of infarction. On the other hand, serious complications of temporary pacing include infection, pneumothorax, air emboli, and cardiac tamponade. However, there have been few reports of such complications leading to interrupted pacing or death in children [31]. In this study, pediatric patients treated with ECMO had more side effects than those treated with temporary pacing (*P* < 0.05). Therefore, in pediatric patients with acute myocarditis undergoing emergency intervention, we propose avoiding unnecessary ECMO treatment due to its possible side effects. 

### Strengths and limitations

Our study has several limitations. It was a single-center retrospective study with a relatively small number of patients. In addition, a myocardial biopsy was not performed. However, our study might be valuable because there are very few studies focusing on the relationship between disease severity, and wide QRS complexes with ST-segment changes on admission. Large-scale prospective studies are required to validate these findings.

## Conclusion

In pediatric patients with acute myocarditis undergoing emergency interventions, it is essential to classify ECMO or temporary pacing in terms of therapeutic efficacy and complications based on ECG findings on admission. In patients with wide QRS complexes with ST-segment changes on admission, ECMO is required immediately. However, temporary pacing could be effective in patients with CAVB and/or advanced AVB and narrow QRS, and ECMO might not be necessary.

It is essential to promptly administer ECMO in patients with wide QRS complexes with ST-segment changes on admission. Conversely, patients with narrow QRS without circulatory collapse could improve without ECMO administration. In pediatric patients with acute myocarditis undergoing emergency intervention, ECMO or temporary pacing could be classified based on wide QRS complexes with ST segment changes on admission. Therefore, excessive treatment should be avoided by separating ECMO from temporary pacing.

## Data Availability

All data generated or analysed during this study are included in this published article.

## Abbreviations

AV: Atrioventricular
AVB: Atrioventricular block
CAVB: Complete atrioventricular block
ECG: Electrocardiogram
ECMO: Extracorporeal membrane oxygenation
LVEF: Left ventricular ejection fraction
